# Understanding Nociplastic Pain: Building a Bridge between Clinical Psychology and Medicine

**DOI:** 10.3390/jpm13020310

**Published:** 2023-02-10

**Authors:** Federica Galli

**Affiliations:** Department of Dynamic and Clinical Psychology and Health Studies, “Sapienza” University of Rome, Via degli Apuli 1, 00185 Rome, Italy; f.galli@uniroma1.it

Chronic pain (CP), defined as pain lasting more than three months, is a significant healthcare challenge with considerable economic costs. Prevalence rates of CP are between 11% and 40% [1] A systematic review reported a pooled CP prevalence rate of 43.5%, with the rate of moderate-to-severe disabling pain ranging from 10% to 14% [2]. CP prevalence increases with age and is greater among females and people with lower socioeconomic status [3,4]. CP affects relationships and self-esteem and is associated with higher divorce and suicide rates and an increased risk of substance abuse [5], psychopathology [6], and medication overuse [7,8]. The causes of CP are still poorly understood. The reasons why a natural protective factor such as acute pain becomes a disabling and often everlasting condition is a matter of scientific debate. Recently, the International Association for the Study of Pain [9] has proposed that three subtypes of CP (nociceptive, neuropathic, and nociplastic) may be differentiated based on unique causal mechanisms. Accordingly, nociplastic pain (NP) is a new descriptor of CP and includes conditions that arise from altered nociception despite no clear evidence of actual or threatened tissue damage. This new descriptor attempts to characterize conditions previously known as functional pain syndromes or unexplained pain, relieving patients from the possible stigma of “all in my head” rationalizations. NP should be viewed as an overarching terminology applicable to a diverse range of clinical conditions that share common neurophysiological mechanisms involving various organ systems. Several types of chronic primary headaches and orofacial pain, chronic visceral pain syndromes, chronic widespread pain and fibromyalgia, and chronic primary musculoskeletal pain have been included among nociplastic chronic syndromes [5]. NP is usually accompanied by other CNS-associated symptoms with a close link with clinical psychological factors: general symptoms (e.g., fatigue and cognitive problems), temperamental characteristics (e.g., hypersensitivity to environmental stimuli), and psychopathological symptoms (e.g., anxiety/depression) [5]. The potential role of psychology in this field is clear, both on the side of research and clinical approach. On the one hand, we know that different CP conditions have a history of adverse childhood experiences and comorbid psychopathological milieu [10]. These psychological problems might shift from having a comorbid status to being causative of CP of NP origin by influencing, at some yet unknown level, the nervous system. Research at this level is crucial. A sensitized nervous system has been considered one of the most important mechanisms involved in NP [11]. Central sensitization (CS) is defined as the increased responsiveness of nociceptive neurons in the central nervous system to their normal or subthreshold afferent input, according to the IASP [12]. Many emotional states, such as depression and anxiety, and emotional processes, such as emotional awareness and regulation, influence the presence and severity of NP conditions [13], revealing the importance of a psychological assessment for diagnosis and psychological intervention(s). Highlighting clusters of disorders whose NP component is predominant, mixed, or absent can lead to a personalized clinical approach to the patients, both from a medical and psychological perspective.

In synthesis, the concept of NP establishes a new framework for understanding the co-occurrence of different chronic disorders and the role of related psychological factors. Comorbid disorders may be the expression of shared pathophysiological mechanisms, with etiological and psychological features differentiating them by “non-nociplastic” forms of the same disorder. New pharmacological and psychological therapies may result from a better understanding of NP conditions, as well as the explanation and possible discontinuation of worthless clinical interventions.
